# Reasoning over genetic variance information in cause-and-effect models of neurodegenerative diseases

**DOI:** 10.1093/bib/bbv063

**Published:** 2015-08-05

**Authors:** Mufassra Naz, Alpha Tom Kodamullil, Martin Hofmann-Apitius

**Keywords:** BEL model, Alzheimer’s disease, genetic variants, GWAS, causal reasoning, cause-and-effect

## Abstract

The work we present here is based on the recent extension of the syntax of the Biological Expression Language (BEL), which now allows for the representation of genetic variation information in cause-and-effect models. In our article, we describe, how genetic variation information can be used to identify candidate disease mechanisms in diseases with complex aetiology such as Alzheimer’s disease and Parkinson’s disease. In those diseases, we have to assume that many genetic variants contribute moderately to the overall dysregulation that in the case of neurodegenerative diseases has such a long incubation time until the first clinical symptoms are detectable. Owing to the multilevel nature of dysregulation events, systems biomedicine modelling approaches need to combine mechanistic information from various levels, including gene expression, microRNA (miRNA) expression, protein–protein interaction, genetic variation and pathway. OpenBEL, the open source version of BEL, has recently been extended to match this requirement, and we demonstrate in our article, how candidate mechanisms for early dysregulation events in Alzheimer’s disease can be identified based on an integrative mining approach that identifies ‘chains of causation’ that include single nucleotide polymorphism information in BEL models.

## Systems biology models and genetic variation: two separate worlds

Barabási *et* *al*. [1] assert ‘given the functional interdependencies between the molecular components in a human cell, a disease is rarely a consequence of an abnormality in a single gene, but reflects the perturbations of the complex intracellular and intercellular network’.

Genome-wide genetic association studies (GWAS) have become a useful and frequently used tool for discovering genetic variants as a disease risk [2]. However, for complex traits and phenotypes, interpretation of association data largely benefits from available prior biological and environmental knowledge, spanning over multiple scientific disciplines [3].

In human genetics, several strategies were developed and implemented to determine the effect of single nucleotide polymorphisms (SNPs), particularly, for the analysis of genotyping data. The limitation of many of these algorithms is that they can predict only either to have no effect or to have negative effect on clinical readouts and endpoints. However, the spectrum of possible biological effects caused by genetic variants is much wider, and thus, methods are required to predict also potential gain, loss or even modification of gene function [4]. Moreover, most of the algorithms can predict only variant effects on individual proteins [5], and machine learning supervised and semi-supervised approaches are being used to predict the effect of deleterious SNPs [4]. Generally, GWA studies are used to establish links between genotypes and phenotypes through identifying the differences (and commonalities) between thousands of individuals. These approaches work as black boxes and make use of statistical and machine learning approaches that require huge data sets.

To reveal the functional context at the molecular level, substantial knowledge about the genes involved, their expression at RNA and protein level, the time when they are expressed and in which tissue and in which organ, is required. Regulation of gene expression is mediated through genetic regulatory systems, which are controlled by complex interaction networks involving DNA, RNA, proteins and small molecules. These regulatory networks involve many components linked to each other by positive and negative feedback loops, and a deterministic understanding of their dynamics is hard to attain owing to rapidly increasing complexity. Therefore, specialized methods and computer software are essential, for the modelling and simulation of genetic regulatory networks [6].

Systems biology is the systemic contextual representation and modelling of a plurality of discrete observations. In systems biology, modelling is a representation of disease high-level concepts in a unified and comprehensive network that can help to identify the differential subnetworks by comparing it with a network representing the healthy state [1, 7–10]. Building blocks of systems biology models, such as signalling pathways, metabolic systems and gene regulation networks, are already widely used in computational biology. Comprehensive disease models, however, are going way beyond these comparably well-understood functional modules. One of the explicit goals of systems biomedicine is to use a generalized model of disease to assess the parameters from high-throughput data of a single patient, to generate ‘personalized models’ that predict disease progression and treatment responses [11, 12].

In systems biology, there are several ‘entry points’ to generate initial networks: protein–protein interaction, metabolic networks and signalling pathways have been widely used to model biological processes [7]. In the past decade, however, new modelling approaches have been developed [13]. For pharmacogenomics, these networks represent complex relationships between drugs and targets. The diseasome [14] is a disease–gene and drug–target (protein) network, where disease information is associated with a gene and drugs are linked to proteins by drug–target associations [15].

Despite the complexity of regulatory networks, attempts at unravelling the impact of genetic variation on regulatory networks have been addressed by a number of groups. Leiserson *et al*. [16], Carter *et al*. [17] and Atias *et al*. [18] have worked on network approaches to scrutinize the genetic risks for human disease. They have developed methodology that allows to detect causal genes within disease-associated loci by network analysis, and to ascertain causal paths from allele to disease through intermediate molecular phenotypes [16–18]. Trynka *et al*. [19] proposed new approaches on the interpretation of transcriptional regulation effects to estimate the involvement of variant alleles in common diseases. They suggested that most of the causal complex trait variants have regulatory roles with cell type specificity, by interconnecting GWAS data with genome-wide chromatin assays results. They emphasized the importance of cell-type-specific regulatory context and highlighted the value of the inclusion of epigenomics information [19].

Sahni *et al*. [20] questioned the strong bias in the literature towards coding variant effects on protein–DNA, protein–RNA and protein–protein interactions. He proposed to put more emphasis on effects outside the protein centric scope of functional assessment, to understand the impact of genetic variants on specific interactions; for instance, mechanisms safeguarding protein folding and stability [20].

## Types of genetic variation information relevant for systems biomedicine

If genetic variation is to be included in a systems biology model of disease, we need to assess the biological impact of a SNP or a mutation. Dependent on the way (the ‘mode-of-action’) how a SNP or a mutation exerts its biological impact, we can distinguish several classes (‘types’) of SNPs. In this section, we identify and discuss the different functional categories that can be distinguished as ‘mode-of-SNP-action’ classes (see Table 1).

**Table 1.** Types of genetic variation information relevant for systems biomedicine: DNA regions with functional categories and consequences

Types of genetic variation information relevant for systems biomedicine		
DNA regions	Functional categories	Functional consequences
1. Coding regions	1. Non-synonymous genetic variants	Change in protein structure or function due to a change in the amino acidsequence or protein sequence truncation
	2. Synonymous genetic variants	Modulating translation rates with direct consequences to protein folding
	3. Exon splicing enhancers or silencers	Translate the protein isoform by deleterious intron retention or exon skipping
2. Non-coding regions	1. DNA methylation	Associates with genes silencing
	2. Transcription factor binding to regulatory elements	Can change transcription factor binding to DNA that leads to differential target gene expression
	3. Chromatin loop bridging the enhancers and promoters	Can alter the DNA affinity for looping factors and chromatin interactions, which regulates gene expression
	4. MiRNAs	Can affect gene functionality: (i) by transcription of primary transcript, (ii) by pri-miRNA and pre-miRNA processing and (iii) by effecting miRNA–miRNA interaction
	5. lncRNAs	Can modify highly conserved lncRNA tertiary structure that can affect chromatin regulator’s interactions

### Genetic variants on coding regions

The risk associated with non-synonymous genetic variants can be easily translated into a change in protein structure or function owing to a change in the amino acid sequence. It can modify amino acid composition, or truncate the protein sequence by causing an early stop codon [21]. Synonymous genetic variants do not alter the codon sequence. However, synonymous genetic risk variants can still impact protein function by modulating translation rates with direct consequences to protein folding [22]. For example, rs1045642 SNP slows down the rate of translation of the MDR1 mRNA and impacts protein folding [23]. Exon splicing enhancers or silencers are typically 6–8 consecutive nucleotide sequences in an exon region. Where, SNP can also result in deleterious intron retention or exon skipping, and translate the protein isoform [24–27]. For example, rs1800693 SNP affects the splicing of the TNFRSF1A mRNA, leading to translate an isoform [28].

### Genetic variants on non-coding regions

Model gene system studies have revealed that local DNA interactions between regulatory sites and genes are important for transcriptional control. Such regulatory interactions, in mammals, can take place over significant chromosomal distances up to an entire mega-base (1 Mb) [29]. Genetic risk variants are frequent on non-coding sequences [30]. Post-GWAS studies have revealed the capacity of these genetic risk variants to regulate gene expression by modulating cis-regulatory machineries through mechanisms involving DNA methylation [31], transcription factor binding [32], chromatin looping [33] or microRNA (miRNA) recruitment [34]. If SNPs occur within transcriptional regulatory regions, like transcription factor binding sites, CpG islands and miRNAs, they may modify the binding affinity of the regions, remove recognition sites or create new binding sites for other regulatory proteins. All of these modifications can lead to alterations in the level, timing and localization of gene expression [35].

#### DNA methylation

DNA methylation means addition of methyl groups to a cytosine nucleotide, which is basically part of a CpG dinucleotide [36]. DNA hyper-methylation near transcription start sites of tumour suppressor genes associates with their silencing [37].

#### Transcription factor binding to regulatory elements

Across the genome, transcription factors bind to thousands of regulatory elements, including promoters (directly upstream of their target genes) and cis-regulatory elements such as enhancers, insulators and silencers [38]. Genetic risk variants located within promoter regions can also change transcription factor binding to DNA, leading to differential target gene expression [39, 40]. For example, expression of the α-globin gene locus is affected by a genetic variant associated with the a-thalassaemia blood disorder [39]. Enhancers are commonly targeted by those genetic variants of risk-associated loci that map to DNA recognition motifs, bound by transcription factors. These genetic variants can modulate the chromatin affinity for transcription factors and consequently gene expression [33, 41–46, 47]. For example, the rs12740374 SNP, which is associated with a lower level of plasma low-density lipoprotein cholesterol, increases the expression level of the SORT1 (Sortilin 1) gene by increasing the binding affinity of the C/EBP (CCAAT enhancer-binding protein) transcription factor to chromatin [33].

#### Chromatin loop bridging the enhancers and promoters

Genetic risk variants can modulate chromatin loop formation; it can alter the DNA affinity for looping factors, which results in allele-specific chromatin loop formation. The human genome is structured in a three-dimensional architecture, which is thought to regulate a diverse set of DNA-template processes [47–52]. This facilitates regulatory elements, like promoters and enhancers, to interact physically through long-range chromatin loops, or chromatin interactions, to regulate gene expression [53, 54]. This has been shown for the rs12913832 SNP, which resides in an enhancer 21 Kb upstream of the OCA2 (Oculocutaneous albinism II) pigment gene [55]. Over the past decade, the development of chromosome conformation capture (3C) technology has initiated several 3D studies on regulatory chromatin loops, but what has been done until now is far from exhaustive. If a minor fraction of these potential regulatory elements participate in chromatin looping, then most of the genomic interactions have yet to be characterized again, because many such loops appear to be tissue-specific [56–58], which makes their comprehensive analysis appear even more disconcerting [59].

#### MicroRNAs

miRNAs target mRNAs by recognizing their complementary sequences mainly in 3′ untranslated regions (3′UTRs). miRNAs largely function as post-transcriptional repressors. They recruit RNA-induced silencing complex to their target mRNAs, leading to mRNA degradation or translation repression [60]. They can regulate the translation of hundreds of genes through sequence-specific binding to mRNA [61]. SNP variants, linked with miRNAs, can affect gene functionality with three different ways: (i) by transcription of primary transcript, (ii) by primary miRNA (pri-miRNA) and precursor miRNA (pre-miRNA) processing and (iii) by effecting miRNA–miRNA interaction [62]. For example, rs10065172, a Crohn’s disease-associated SNP, lies within the 3′UTR of the IRGM (immunity-related GTPase M) gene and alters the complementary target sequence of miRNA-196 [36].

#### Long non-coding RNAs

Long non-coding RNAs (lncRNAs) are found across intergenic regions of the human genome [63]. They can interact with chromatin regulators for their recruitment by chromatin [64, 65], a process that relies on a highly conserved lncRNA tertiary structure, which can be changed by genetic risk variants [66]. Kim *et al*. [67] described enhancer RNAs (eRNAs), a new class of non-coding RNAs, formed from polymerase II-bound enhancers. The level of expression of eRNAs is positively correlated with the expression of neighbouring coding genes [67] Genetic variants in enhancer sequences can modify Transcription Factor (TF) binding, resulting in ‘improper’ gene expression and eventually susceptibility to diseases [68, 69]. The micropeptides, called small pri-peptides, are also expressed from the lncRNA-pri and direct the proteolytic cleavage or other modifications of target proteins or transcription factors [70].

#### Expression quantitative trait loci

Studying the association between genetic variation and gene expression offers a straightforward way to begin the complicated task of connecting risk variants to their putative target genes [71]. Networks created using gene expression data from patient samples can be exploited to bridge GWAS results with an underlying disease mechanism, as exemplified in the autism spectrum disorder [72]. Genetic variation associated with gene expression, known as expression quantitative trait loci (eQTL), can identify the target genes of risk loci [73–77]. Polymorphism situated in DNA regulatory elements can alter the gene transcript frequency. Thus, as a quantitative trait locus, gene transcript frequency can be determined with substantial power [78, 79]. Brem *et al*. [80] published the first genome-wide study of gene expression in 2002. Stranger and Raj [81] reviewed the genetics of human variation and diversity in eQTLs. These eQTL data are dynamic with great specificity for different tissues and environmental perturbations.

### The ENCODE project: identification of genomic functional elements

The ENCODE project has delivered an incredible compilation of genetic functional elements of the human genome [82]. As most of the SNPs detected in GWAS data belong to non-coding regions of the genome, usage of ENCODE regulatory elements to reinterpret GWAS data sets might be a valuable approach [83]. Undoubtedly, structural genomic variation are more influential and systemic than the smaller scale variations; however, any framework or methodology used to predict genetic variant effects needs to contribute for both small- and large-scale variations [13]. If possible, it should be able to predict the level in which coding or non-coding genetic variants individually or collectively have a functional impact on biology, ranging from relevant protein function or expression to the perturbation of entire networks. It can help us to annotate the massive amount of re-sequencing data meaningfully without having to test the effects of all variants experimentally [13].

Thus, now it is the time to move ahead from merely bio-statistical approaches for GWAS data interpretations to a more comprehensive approach that can be acquainted with gene–gene and gene–environment interactions, along with the complexity of the relationship between genotype and phenotype [84].

## The need to integrate genetic variant information in systems biomedicine models

Currently, GWAS variance data interpretation has become a bottleneck in the progression of mapping and exploring complex diseases. For example, multiple genes have been associated with Amyotrophic lateral sclerosis in GWAS data, but there is no clear perspective of involved pathways and mechanisms that would emerge from the available high-throughput data, by taking multiple rare variants into account [85].

Substantial research for several complex diseases has been conducted to unravel causal mechanisms underlying their disease aetiology. Often this type of research is multidisciplinary, using research studies spreading over a wide range of time and length scales. Consequently, a disease model representing disease aetiology may have many modules and interactions. Such a disease model would provide a nice template for the interpretation of the functional consequences of genetic variation [86].

One of the obvious questions is of course, which methodology can help in interpretation of GWAS data, when most of the SNPs have small effects on disease susceptibility [87]. There is lack of efficient and reliable algorithms as well as appropriate multi-scale modelling methodology, to evaluate the huge number of interdependent data from GWAS [5]. One way to reduce the combinatorial complexity of GWAS data is, to reduce the dimensionality of genetic variation data by taking a priori knowledge about functional relationships between genes and proteins into account. Formalized knowledge about causal and correlative relationships in systems biology models provides a good starting point for that dimension reduction. So far, there have been only few serious efforts to predict how these genetic variants would collectively be effective for specific phenotypes [88, 89].

## Systems biology modelling language syntax adaptations

A massive amount of data for molecular interactions and pathways are stored in online databases. Moreover, experimental data are accumulating rapidly, and correspondingly, the demand for exchange of data to allow analysis and comparison of larger data sets is intensifying. Thus, there is a need for representation of data in standardized formats. Comparisons and evaluations of modern systems biology modelling languages show [90,91] that XML is a remarkable and easy-to-use format for systems biology information representation. Here, we compare the recent updates with the standard XML-based representation formats for exchange of data.

### The Resource Description Framework

The Resource Description Framework (RDF) model [92] is based on the idea of making statements about resources. A RDF statement, also called a triple in RDF terminology, is an association of the form (subject, predicate, object). RDF Schema [93] and the Web Ontology Language [94] are used to explicitly represent the meanings of the resources described on the Web and how they are related. These specifications, called ontologies, describe the semantics of classes and properties used in Web documents. These ontologies should be linked to a top-level ontology to enable knowledge sharing and reuse [94]. Unfortunately, each bio-ontology seems to be built as an independent piece of information, which does not enable the sharing and reuse of knowledge and complicates data integration [95]. Moreover, various sources of biological data must be combined to obtain a full picture and to build new knowledge. However, a large majority of current databases does not use a uniform way to name biological entities. As a result, a same biomedical object is frequently associated with different names.

### Systems Biology Markup Language

Systems Biology Markup Language (SBML) [96–99] was designed by the Systems Biology Workbench Development group. The purpose of SBML is to model biochemical reaction networks, comprising cell signalling, gene regulation and metabolic pathways. In SBML, ‘Species’ is used as a notation to represent the interactors, while reaction, modelling a transformation, transport or binding to represent interaction. Each reaction is allowed to interact with three predefined interactors i.e. reactant, product and modifier [100]. An SBML model encodes a reaction network as pathway. Mathematical relations are also available for reactions. References to other sources and extra information can be added only in the annotation field. Currently, the representation of parts of molecules is not possible [101].

### The Proteomics Standards Initiative Molecular Interaction XML format

The Proteomics Standards Initiative Molecular Interaction XML format (PSI MI) [102] is designed by the Proteomics Standards Initiative, which is an initiative of the Human Proteome Organization. The main purpose of the initiative is to standardize proteomics data representation to facilitate data exchange, comparison and verification. The format is projected for exchange of protein–protein interaction data [102]. PSI MI is structured around an entry. It is not anticipated to be a pathway [101]. Links to publications and databases are possible, but a representation of relationships through mathematical equations and an inheritance is not available [101].

### The Biological Pathway Exchange

The Biological Pathway Exchange (BioPAX) format is designed by the BioPAX working group [103, 104]. The main purpose of this standard is to introduce a unified framework for sharing pathway information. BioPAX offers more explicit use of relations between concepts than SBML and PSI MI. It is defined as ontology of concepts with attributes [104]. However, reasoning and integration of data increases its computational complexity [101]. A specific data type is available for pathway representation, but mathematical equations underlying the relations are not possible.

### CellML

CellML Model Repository [105] contains biochemical pathway models that have been published in peer-reviewed articles or expressed in SBML [106]. CellML [107] and the CellML Model Repository are part of the IUPS Physiome Project [108]. The CellML Model Repository contains models describing a wide range of biological processes [109]. It uses mathematical descriptions of biological systems and adds semantic meaning by annotating elements by ontologies and constrained vocabularies [109]. It is also precise, and thus, the association between dependent and independent species is implicit rather than explicit. However owing to this generality and explicit nature, complexity is increased, especially for software developers, and consequently, there are a few tools that can read and write CellML [110].

### Biological Expression Language

Biological Expression Language (BEL) is a highly expressive, triple-based knowledge representation language for the representation of knowledge about causal and correlative relationships [111]. Several groups in academia and pharma are already applying BEL in various areas including biological network analysis, disease modelling, understanding drug efficacy and toxicity, mechanisms for drug sensitivity and resistance and other research and development-related projects. A suite of software components called the BEL Framework provides tools that are required to create, compile, assemble and deliver computable knowledge models to BEL-aware applications [111].

BEL represents complex biological content as simplified, formalized, computable semantic triples that provide the ability to use and reuse experimental observations. BEL can also be used for next-generation sequencing applications, like gene expression profiling and genome annotation data, by using Reverse Causal Reasoning (RCR) algorithm to get mechanistic insights into the high-throughput data, which could be complementary to the result of analysis using pathway gene set. BEL has many utility tools such as a dedicated Cytoscape plug-in for network visualization, algorithms of causal reasoning (RCR) for understanding disease mechanism by identifying up-stream and down-stream controllers, electronic workbook integration, BEL-to-RDF translation, text mining in BEL and nano-publication concepts [112]. BEL has the potential to impact scientific literature, by introducing computable expressions in scientific publishing, which could be integrated efficiently into existing knowledge environment [113]. Moreover, these causal-reasoning models can provide a valuable addition to the biologists to interpret the gene expression data [114]. By using these models, Huang *et al*. [115] has proposed a data-driven method, Correlation Set Analysis, to detect active regulators in disease by integrating co-expression analysis and literature-derived causal relationships [115].

## Reasoning over genetic variance information integrated in disease networks: concepts and strategies

A key task in genetic variants interpretation, to understand the phenotypic consequences, lies in the ability to predict the molecular-level mechanistic consequences of gene polymorphisms and mutations.

As a consequence, systems biomedicine modelling approaches need to combine mechanistic information from various levels, including gene expression, miRNA expression, protein–protein interaction, genetic variation and pathway information. OpenBEL, the open source version of BEL, has recently been extended to match this requirement. With the extended syntax, the new version of BEL 2.0 is now enabled for encoding genetic variants in biomedical models. The last release of the BEL syntax proposes a representation for different genetic variant types, for example, <substitution>, <insertion>, <deletion> and <intergenic>, by introducing new variant functions for DNA, RNA and protein levels.

In this version, the variant (<expression>) function can be used as an argument within a gene(), rna(), microRNA() or protein() to indicate a sequence variant of the specified level. The variant() function takes Human Genome Variation Society (HGVS) variant description expression, e.g. for a substitution, insertion, or deletion of variants. The extended BEL syntax is supposed to support reasoning over cause-effect models that include genetic variation information.

## Representation of variant at protein level

Effects of genetic variants located on coding region or splice site, if expressed at protein level, can be represented through protein-level functions. Protein-level variants representation is purposed to see the genetic variants with their relevancy to protein, like their location on the protein sequence and effect on the protein structure (see Table 2).

**Table 2.** Representation of different genetic variant categories with variant functions at proteins level in BEL (2.0V)

Variant categories	Variant() function in protein
Reference allele	p(HGNC:CFTR, var(=))
Unspecified variant	p(HGNC:CFTR, var(?))
Substitution variant	p(REF:NP_000483.3, var(p.Gly576Ala))
Deletion variant	p(REF:NP_000483.3, var(p.Phe508del))
Frameshift variant (HGVS short description)	p(REF:NP_000483.3, var(p.Thr1220Lysfs))
Frameshift variant (HGVS long description)	p(REF:NP_000483.3, var(p.Thr1220Lysfs*7))

## Representation of variant across DNA/RNA

To see the genetic variants’ impact at DNA/RNA level, protein-level variants can also be expressed by DNA/RNA-level functions. Whereas, genetic variants located on non-coding regions (like intergenic or intronic) can only be represented through DNA/RNA-level functions, which are designed to see the genetic variants with their relevancy to genome or gene expression (see Table 3).

**Table 3.** Representation of genetic variants across DNA/RNA with the reference of chromosomal or mRNA position in BEL (2.0V)

Level categories	var() function at different genetic levels
DNA—SNP	g(SNP:rs113993960, var(delCTT))
DNA—chromosome	g(REF:NC_000007.13, var(g.117199646_117199648delCTT))
DNA—coding sequence	g(REF:NM_000492.3, var(c.1521_1523delCTT))
RNA—coding sequence	r(REF:NM_000492.3, var(c.1521_1523delCTT))
RNA—RNA sequence	r(REF:NM_000492.3, var(r.1653_1655delcuu))

## Integration of genetic variation information in BEL models of Alzheimer’s disease: enhanced functional interpretation of complex SNP patterns

As a support of this review, here we demonstrate an example to highlight this promising approach, by integrating genetic variant information into an Alzheimer’s disease (AD) BEL model.

We have recently published the AD BEL model [116]. This model has 4052 nodes and 9926 edges, and it was generated by extracting relevant knowledge from the specific biomedical literature. The AD BEL model comprises disease-associated genes, protein–protein interactions, miRNAs, bioprocesses and pathways. To integrate disease-specific genetic variant information into AD BEL model, genetic data are retrieved from GWAS databases and the biomedical literature using text-mining methods. The AD BEL model was enriched with AD-SNP-associated data, after annotating functional impact of these genetic variants using the ENSEMBL variant database.

Subsequently, these genetic variants were prioritized, according to their functional consequences. Then we mapped them to the AD BEL model to identify subnetworks with SNPs that display a substantial biological impact. To complete the functional impact assessment for these variants, we have excavated the biomedical literature to analyse the role of these SNPs in the context of age of onset of AD and specifically in the endocytosis pathway.

The early endosome is the first vacuolar compartment in the context of EP, where enlarged early endosomes are identified as the earliest neuro-pathologic features to develop in the early onset of AD. In sporadic AD, endosomal enlargement adds to an average 2.5-fold larger total endosomal volume per neuron, suggesting a significant increase in endocytic activity. It is the site of internalization and initial processing of amyloid precursor protein (APP) and apolipoprotein E, two significant proteins in AD aetiology [117–119]. Here we focus on the internalization of APP based on the functional role of SNPs.

AD is mainly characterized by the deposition of insoluble amyloid beta peptides 42 (Aβ42) in the brain, which cannot be easily removed through the blood–brain barrier. In healthy brain, APP is processed by ADAM10, which produces soluble amyloid beta peptide 40 (Aβ40), whereas in the non-amyloidogenic pathway, APP is proteolytically processed by BACE and γ-secretase to generate Aβ42 peptides. A SNP rs514049, linked to the ADAM10 gene, may perturb the normal processing of APP to produce soluble Aβ40, as rs514049 is associated with lower level of CSF APPα in AD [120]. BACE1 and BACE2 are associated with γ-secretase complex proteins. Moreover, a SNP rs3754048, with allele G, in the promoter of APH1A gene, might alter the binding ability of YY1 transcription factor, resulting in an increased level of APH1A and γ-secretase activity to facilitate Aβ42 generation [121].

All these players in the non-amyloidogenic pathway are trans-membrane proteins, which traffic through the endocytic pathway [122], where these proteins are internalized from the plasma membrane and recycled back to the surface (as in early endosomes and recycling endosomes), or, alternatively, sorted to degradation (as in late endosomes and lysosomes [123, 124]. However, BACE1 is a genetically significant gene with a number of high-ranked AD-associated SNPs. It is also evident that APP and BACE1 are up-regulated in AD. Moreover, experimental evidence suggested that at the cell surface, APP and BACE1 strongly interact and co-localize and are being internalized together into early endosomes, where both proteins remain co-localized and produce amyloid-β. This evidence confirms that endocytosis may be an important step for amyloid-β production [125]. This can be again supported by the association of genetic variants linked with the trafficking proteins in the EP.

**Figure 1.**
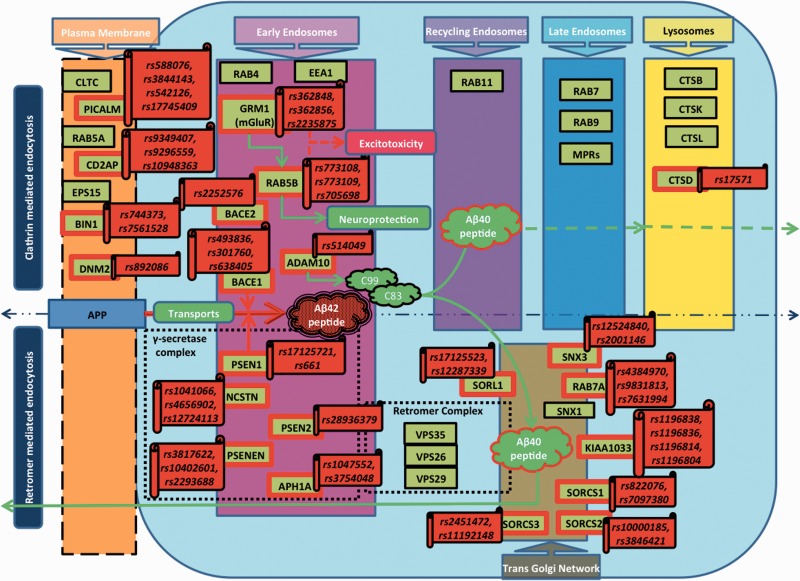
In this diagram, we present a flowchart that depicts an abstracted BEL subnetwork derived from the original AD BEL Model. This flowchart represents causal relationships between genes and genetic variants for the EP components. Gene symbols written in the textboxes with red outline are showing association with the GWAS identified SNPs for AD. A colour version of this figure is available at BIB online: http://bib.oxfordjournals.org.

As shown in Figure 1, there are two branches of EP: firstly, clathrin-mediated endocytosis (CME), and secondly, retromer-mediated endocytosis. In the CME pathway, various proteins such as CLTC, PICALM, DNM2, EPS15 and BIN1 modulate APP transport for its further internalization, subsequent Aβ generation and further processing in lysosomes, which is required for neurotransmission and signal transduction. CLTC is a major protein component of coated vesicles and coated pits in CME pathway [126]. These specialized organelles are involved in the intracellular trafficking of receptors and endocytosis of a variety of macromolecules including APP with the help of additional accessory proteins such as PICALM, EPS1, DNM2, EGF and its substrate EPS15. PICALM encodes a clathrin assembly protein, which recruits CLTC and AP2, and regulates the size of the clathrin vesicle at neuromuscular junction, whereas an intronic PICALM SNP, rs588076, is associated with allelic expression of a PICALM isoform [127]. Stable DNM2 recruitment during CME correlates well with CLTC lifetime [128], while a risk allele at rs892086 is associated with reduced expression of DNM2 mRNA in the hippocampus in AD patients compared with non-demented controls [129].

On the other hand, the EP is also regulated by retromer, which transports APP from early endosomes to trans-Golgi network (TGN) and released outside cell mainly by retromer complex (VPS35, VPS29, VPS26), SORL1, SNX3, SNX1, WASH complex (KIAA1033) and so on [130]. SORL1 protein belongs to type-I trans-membrane, which is expressed in neurons and plays a critical role in the intracellular transport and in APP processing. SORL1 binds to the retromer complex and works as an adaptor protein for APP trafficking from endosomes to TGN. It is observed that SORL1 levels are reduced in AD-diseased brain, while overexpression of it redistributes APP to the Golgi apparatus; thus, the placement and interaction time of APP and BACE1 is reduced in the early endosomes, which will reduce the amount of Aβ42. SNX3 mediates recruitment of cargo-selective retromer complex in association with VPS35 [130]. Recent studies have shown that SNX3 and RAB7A are also required for proper recruitment of the cargo-selective complex. Constitutively active RAB7A Q67L mutant is overexpressed, resulting in displacement of the cargo-selective complex [131]. The cargo-selective retromer subcomplex (VPS35–VPS29–VPS26) recruits the WASH complex (KIAA1033), which mediates the production of branched actin networks on the surface of endosomes. The cargo-selective retromer complex together with SNX27 and the WASH complex operates in the endosome-to-cell surface recycling of receptors and proteins.

## Integration of genetic variation information enhances the evidence base for shared pathophysiology pathways in neurodegenerative diseases

Parkinson’s Disease (PD) and AD may share pathophysiological mechanisms and—as a consequence—may share some of their molecular aetiology. To identify evidence that would speak for shared pathophysiology between AD and PD, we systematically analysed genetic variation information that is common between AD and PD and that can be mapped to putatively shared pathways. We have selected the common SNPs from AD and PD GWAS data, and mapped them to gene annotation. Then we searched diseased BEL models to identify the functional impacts of these genes on AD, PD or neurodegenerative diseases (see Table 4).

**Table 4.** A list of common SNPs/genes in AD and PD with their possible role in the disease context specifically for AD and PD and generally for neurodegenerative diseases (NDD)

Common SNPs in AD and PD	Gene	AD	PD	NDD
rs931977 (Intronic)	ERG2	EGR2 targeted by mAChRs (muscarinic acetylcholine receptors), which is associated with cognitive functions, synaptic plasticity and memory	–	EGR2 is involved in myelination of peripheral nerves
		EGR2 also associated with apoptosis		
rs2672893 (Intronic)	RPTOR	RPTOR is downstream of MTOR and is expressed highly in AD hippocampus	Alpha-synuclein reduced the activation of AMPK target RPTOR	–
		RPTOR activates of PI3K-Akt pathway		
rs6488270 (Intergenic)	Downstream_variant for: TMEM52B	–	GABARAPL1 plays role in development and homeostasis of the mouse brain	GABARAPL1 presents a regulated tissue expression and is the most highly expressed gene among the family in the central nervous system
	Upstream_variant for: GABARAPL1			
rs4742095 (Intergenic)	Upstream_variant for: CD274 PLGRKT	PD1/PD-L1 (CD274) pathway have role in neuroinflammation of AD	–	PLGRKT is regulating plasminogen activation, which plays a key role in regulating catecholaminergic neurosecretory cell function
		PD1/PD-L1 (CD274) pathway is associated with IL-10 production		
				PLGRKT is also involved in macrophage recruitment in the inflammatory response
				PLGRKT is believed to have role in plasminogen binding and cell migration
rs1984129 (Intergenic)	Downstream_variant for: GBP6	–	–	LRRC8B is implicated in proliferation and activation of lymphocytes and monocytes
	Upstream_variant for: LRRC8B			
rs10515758 (Intergenic)	Downstream_variant for: EBF1	–	–	EBF1 have role in axonal pathfinding
	Upstream_variant for: CLINT1			CLINT1 interacts with clathrin, the adapter protein AP-1 and phosphoinositides. This protein may be involved in the formation of clathrin coated vesicles and trafficking between the TGN and endosomes
rs6810871 (Intergenic)	Downstream_variant for: FAM114A1, TMEM156	–	–	FAM114A1 plays a role in neuronal cell development
				FAM114A1 expressed in dentate gyrus, the hippocampus, the cerebellum and the olfactory bulb
	Upstream_variant for: KLHL5, TLR6			

## Conclusion

Given the complexity of neurodegenerative diseases and the limited accessibility to experimental tissues of brain, we need new strategies to integrate data-driven and knowledge-driven approaches to unravel the mechanism behind these complex diseases. Disease networks based on the systems biology models, comprising various interacting molecules such as genes, proteins and bioprocesses, succeeded in integrating most of the available data. In this review, we tried to recapitulate all the major breakthroughs, which demonstrated the collective capturing of disease-related knowledge, modelling it as a system. In addition, we have revisited the major studies around identification of genetic variants and prioritizing these variants based on statistical analysis.

So far, disease networks could not easily accommodate information on genetic variation. We have introduced a novel methodology based on BEL, which enables us to integrate genetic variation information into a disease network. We developed a strategy to analyse the functional consequences of SNPs based on their location in the genome and an interpretation of their putative role in a network model. Currently using the capabilities of extended BEL version, we have developed the AD BEL models together with genetic variants with their DNA, RNA or protein position, variant type and associated allele, which can be used to better understand the role of SNPs in a disease context and tried to predict its consequences based on the functional context provided by the network model.

Although BEL provides certain powerful algorithms like RCR, which allows identifying upstream controllers of an observed effect, there are still limitations to overcome to enable reasoning over genetic variants. It is obvious, that we need to develop more sophisticated algorithms for reasoning over genetic variant information in network models, by integrating the functional impact of genetic variants on genes in the disease context. One route to go to refine that algorithm is based on machine learning approaches to train a model with the established knowledge of functionally identified genetic variants for different complex diseases. That model will then be applied to neurodegenerative diseases to overcome the deficiency of genetic variant evidential data in this area.

Key Points

- Systems biomedicine modelling approaches need to combine various types of mechanistic details to address multilevel nature of disease dysregulation processes.
- This work represents genetic variation information integration in cause-and-effect models to identify candidate disease mechanisms in diseases with complex aetiology.
- It is an integrative mining approach that identifies ‘chains of causation’ with reasoning over genetic information in BEL models.
- It exemplifies a new strategy to integrate data-driven and knowledge-driven approaches to unravel the mechanism of complex diseases.

## Supplementary Data

Supplementary data are available online at http://bib.oxfordjournals.org/.

## Funding

The research leading to these results has received support from the EU/EFPIA Innovative Medicines Initiative Joint Undertaking under AETIONOMY grant agreement n°115568, resources of which are composed of financial contribution from the European Union's Seventh Framework Programme (FP7/2007-2013) and EFPIA companies' in kind contribution.

## Supplementary Material

Supplementary Data
