# Supplementary material for: Reasoning over genetic variance information in cause-and-effect models of neurodegenerative diseases
Source: Brief Bioinform. 2015 Aug 5;17(3):505–16. doi: 10.1093/bib/bbv063 (PMC4870396; doi:10.1093/bib/bbv063)
Supplement: Supplementary Data [file supp_17_3_505__index.html]

Reasoning over genetic variance information in cause-and-effect models of neurodegenerative diseases — Supplementary Data 

# Reasoning over genetic variance information in cause-and-effect models of neurodegenerative diseases

## Supplementary Data

files

- Supplementary Data - xlsx file
- Supplementary Data - docx file
