# Supplementary material for: Reasoning over genetic variance information in cause-and-effect models of neurodegenerative diseases
Source: Brief Bioinform. 2015 Aug 5;17(3):505–16. doi: 10.1093/bib/bbv063 (PMC4870396; doi:10.1093/bib/bbv063)
Supplement: Supplementary Data [file supp_bbv063_Supplementary_BEL_Example.docx]

**Practical details for using BEL as an integrative system biology approach**

We provide here with an example how to interpret the biological impact of a SNP step by step using BEL:

| **Alzheimer’s disease** | **Normal state** |
| --- | --- |
| **BEL codes** | |
| **SET** Citation ={"PubMed", "Brain Res Rev. 2009 Oct;61(2):89-104. doi: 10.1016/j.brainresrev.2009.05.007. Epub 2009 Aug 3.","19651157"}  **SET** Evidence ="An alternative splicing can produce a variant isoform of clusterin which can be translocated to nuclei where it induces apoptosis."  **tloc**(**p**("CLU,isoform"), MESHCL:"Extracellular Space", MESHCL:"Nucleus") -> **bp**(GO:"neuron apoptotic process")  ########################################  **SET** Citation ={"PubMed", "Transl Psychiatry. 2011;1. pii: e18. doi: 10.1038/tp.2011.17.","21892414"}  **SET** Evidence= "We have shown that the minor allele of rs9331888, previously associated with increased risk of AD, is associated with increased-relative levels of NM_203339 and is likely the functional variant responsible for this effect. Given the prior genetic association results for this SNP and the distinct roles of CLU transcripts, 18 we hypothesize that alternative splicing is the etiological link between rs9331888 and AD."  **g**(dbSNP:rs9331888) -> **p**("CLU,isoform")  ##########################################  **SET** Citation={"PubMed","Transl Psychiatry. 2011 Jul 1;1(7). pii: e18","21892414"}  **SET** Evidence="These results suggest a biological mechanism for the genetic association of CLU with AD risk and indicate rs9331888 is one of the functional DNA variants underlying this association."  **p**(HGNC:CLU) positiveCorrelation **path**(MESHD:"Alzheimer Disease")  **g**(dbSNP:rs9331888) positiveCorrelation **path**(MESHD:"Alzheimer Disease")  **g**(HGNC:CLU) -- **g**(dbSNP:rs9331888)  ################################# | **SET** Citation ={"PubMed", "J Alzheimers Dis. 2013;33 Suppl 1:S87-100.","22751174"}  **SET** Evidence="The efficiency of Aβ clearance from brain interstitial fluid across the BBB is influenced by Aβ binding transport proteins such as apoE and apoJ (clusterin), and BBB receptors such as LRP1, LRP2 and receptor for advanced glycation end products (RAGE) which control Aβ efflux from brain and influx into the brain, respectively, and Aβ degrading enzymes"  **SET** NervousSystem= "Brain"  **tport**(**p**(HGNC:CLU)) -> **complex**(**p**(HGNC:CLU),**a**("Amyloid beta-Peptides"))  **tloc**(**a**("Amyloid beta-Peptides"), MESHCL:"Brain", MESHCL:"Blood") -> **deg**(**a**("Amyloid beta-Peptides"))  **deg**(**a**("Amyloid beta-Peptides")) -\| **a**("Amyloid beta-Peptides")  #################################  **SET** Citation ={"PubMed", "Brain Res Rev. 2009 Oct;61(2):89-104. doi: 0.1016/j.brainresrev.2009.05.007. Epub 2009 Aug 3.","19651157"}  **SET** Evidence ="Clusterin is a stress-induced chaperone which is normally secreted but in conditions of cellular stress, it can be transported to cytoplasm where it can bind to Bax protein and inhibit neuronal apoptosis.  "  **tloc**(**p**(HGNC:CLU), MESHCL:"Extracellular Space", MESHCL:"Cytoplasm") -> **complex**(**p**(HGNC:CLU),**p**(HGNC:BAX))  **complex**(**p**(HGNC:CLU),**p**(HGNC:BAX)) -\| **bp**(GO:"neuron apoptotic process") |
| **BEL Network** | |
| 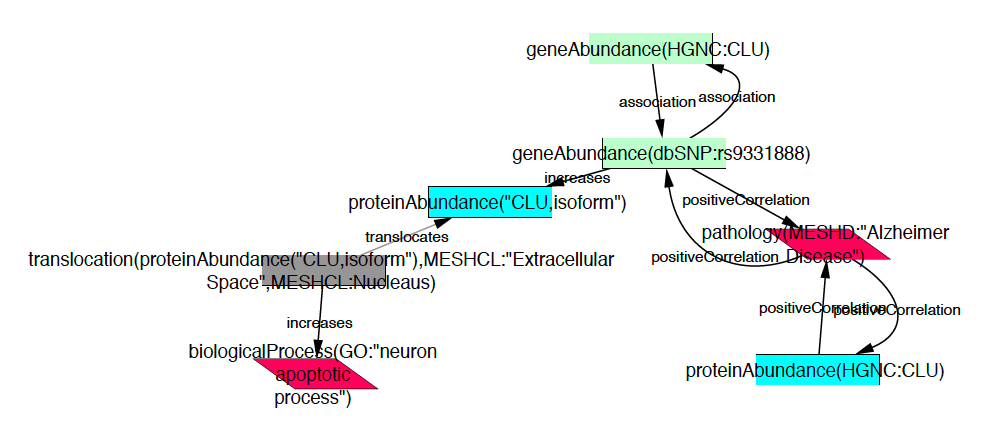 | 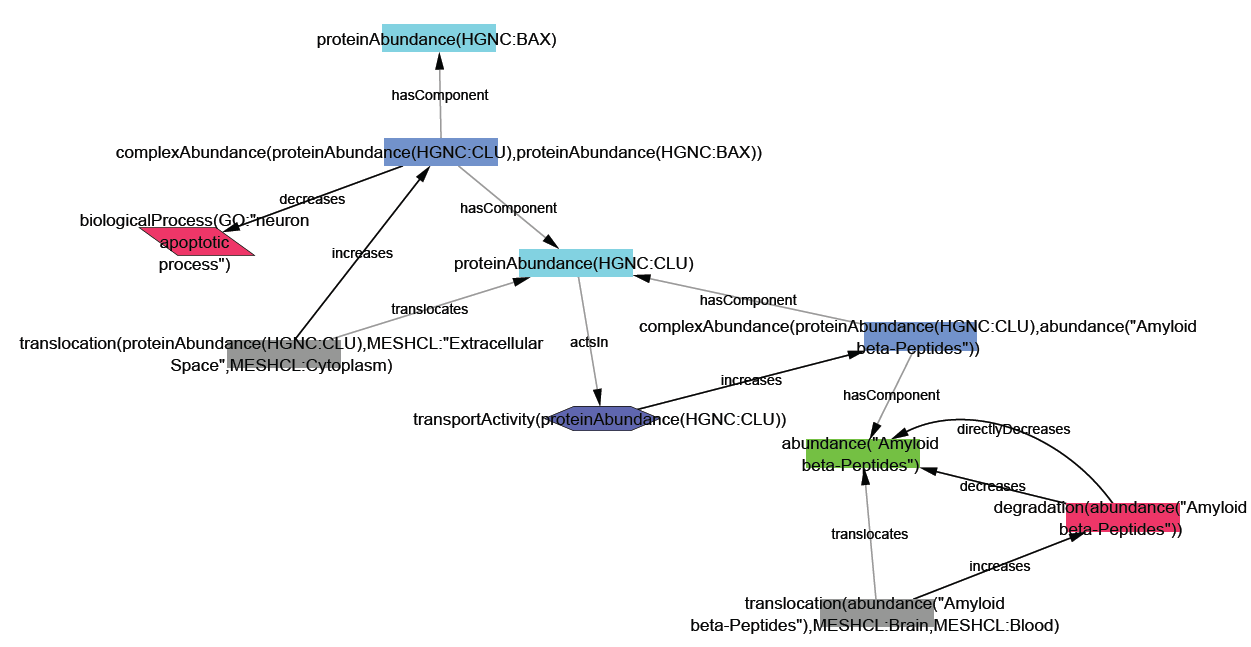 |
| **Chains of causation** | |
| **p**(HGNC:CLU) positiveCorrelation **path**(MESHD:"Alzheimer Disease")  CLU has positive correlation with Alzheimer’s disease  **g**(dbSNP:rs9331888) positiveCorrelation **path**(MESHD:"Alzheimer Disease")  Genetic variant rs9331888 has positive correlation with Alzheimer’s disease  **g**(HGNC:CLU) -- **g**(dbSNP:rs9331888)  CLU is associated with genetic variant rs9331888  **g**(dbSNP:rs9331888) -> **p**("CLU,isoform")  Genetic variant rs9331888 increases CLU isoform  **tloc**(**p**("CLU,isoform"), MESHCL:"Extracellular Space", MESHCL:"Nucleus") -> **bp**(GO:"neuron apoptotic process")  CLU isoform translocate into nucleus and increases neuronal apoptotic process | **tport**(**p**(HGNC:CLU)) -> **complex**(**p**(HGNC:CLU),**a**("Amyloid beta-Peptides"))  Transport activity of CLU increases the complex of CLU and Amyloid beta-Peptides.  **tloc**(**a**("Amyloid beta-Peptides"), MESHCL:"Brain", MESHCL:"Blood") -> **deg**(**a**("Amyloid beta-Peptides"))  Translocation of Amyloid beta-Peptides from brain to blood increases the degradation of Amyloid beta-Peptides.  **deg**(**a**("Amyloid beta-Peptides")) -\| **a**("Amyloid beta-Peptides")  Degradation of Amyloid beta-Peptides deceases Amyloid beta-Peptides accumulation.  **tloc**(**p**(HGNC:CLU), MESHCL:"Extracellular Space", MESHCL:"Cytoplasm") -> **complex**(**p**(HGNC:CLU),**p**(HGNC:BAX))  Translocation of CLU from Extracellular space to Cytoplasm increases complex of CLU and BAX proteins.  **complex**(**p**(HGNC:CLU),**p**(HGNC:BAX)) -\| **bp**(GO:"neuron apoptotic process")  Complex of CLU and BAX proteins inhibits neuron apoptotic process |
| **Cartoon** | |
| 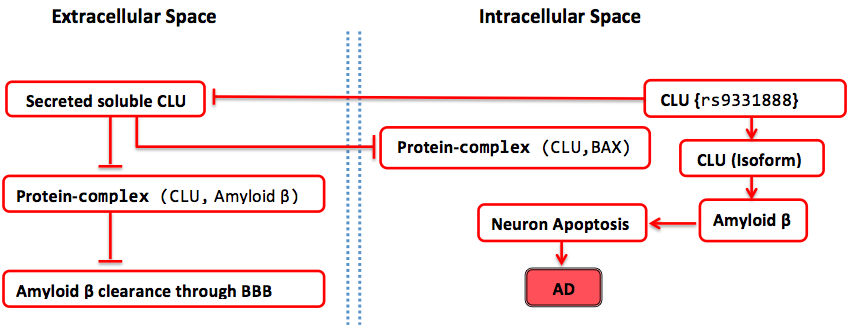 | 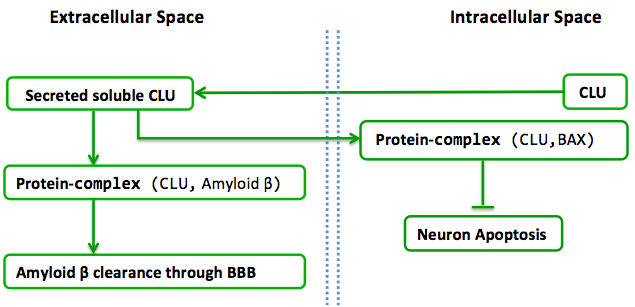 |
| **Impact assessment** | |
| Intronic SNP of CLU gene is found to be associated with the AD disease. The risk variant rs9331888 (with allele G) associated with the CLU gene increases the quantity of a CLU isoform in AD, which induces apoptosis and may contribute to the accumulation of amyloid beta in AD. It is possibly linked to fibrillar amyloid-beta and apoptotic mechanisms in neurodegenerative diseases. | Clusterin (CLU) forms a complex with Aβ. Complex of CLU and Aβ40- CLU is taken up at the BBB. CLU is a transport protein and has a role in helping clearance of amyloid-beta by transporting it through the blood-brain barrier. CLU is normally secreted but in cellular stress condition, it can be moved to cytoplasm where it bind to Bax protein and inhibit neuronal apoptosis. |
